# Quantitative Retinal Vascular Imaging Using Flood-Illuminated Adaptive Optics in Eyes with Lens Opacities

**DOI:** 10.3390/bioengineering13080852

**Published:** 2026-07-23

**Authors:** Yash Porwal, Kenaan Elbash, Marlon Diaz, Emily Butler, Saige Oechsli, Laurence Magder, Andrew J. Bower, Osamah Saeedi

**Affiliations:** Department of Ophthalmology and Visual Sciences, University of Maryland, Baltimore, MD 21201, USA; yporwal@som.umaryland.edu (Y.P.); kelbash@som.umaryland.edu (K.E.); marlon.diaz@som.umaryland.edu (M.D.); emily.butler@som.umaryland.edu (E.B.); soechsli@som.umaryland.edu (S.O.); lmagder@som.umaryland.edu (L.M.); abower@som.umaryland.edu (A.J.B.)

**Keywords:** adaptive optics, flood illumination, retinal vasculature, experiment reproducibility, cataract, lumen diameter, vessel wall, vascular imaging

## Abstract

Flood-illuminated adaptive optics (AO) imaging enables high-resolution quantification of retinal vascular morphology; however, its repeatability in older adults with lens opacities remains poorly characterized. In this prospective study, 40 subjects (66 eyes) underwent flood AO imaging using the rtx1 system (Imagine Eyes, Orsay, France) at three retinal locations across multiple sessions. Two independent masked graders measured lumen diameter (LD), total diameter (TD), wall cross-sectional area (WCSA), and wall-to-lumen ratio (WLR), with repeatability assessed using intraclass correlation coefficients (ICC). Inter-grader agreement was excellent for LD and TD across all regions (ICC range 0.974–0.994) and for WCSA at the superior and inferior regions (ICC range 0.902–0.952). Intrasession and intersession repeatability were similarly excellent for LD and TD across all locations. Repeatability remained excellent across age groups and cataract grades. Image region was the only variable significantly associated with image quality (*p* < 0.0001), with temporal images showing the lowest proportion of good quality ratings (12%). These findings support flood AO imaging as a reliable tool for quantitative vascular assessment in age-related and vascular-related ocular diseases.

## 1. Introduction

Adaptive optics (AO) is a technology that corrects for the eye’s optical aberrations, enabling high-resolution in vivo retinal imaging when integrated with ophthalmic imaging platforms such as scanning laser ophthalmoscopes and optical coherence tomography [1]. This allows for precise assessment of retinal vascular morphology through metrics such as lumen diameter, vessel wall thickness, and more. AO-based vascular metrics have been increasingly used to study vascular alterations associated with aging and ocular diseases such as glaucoma and diabetic retinopathy [2,3,4]. AO-based vascular imaging has primarily been performed using adaptive optics scanning laser ophthalmoscopy (AO-SLO) and adaptive optics optical coherence tomography (AO-OCT) [5,6].

The repeatability of AO-derived measurements has been extensively characterized in the context of photoreceptor imaging. Prior work has demonstrated good to excellent repeatability of cone density metrics using AO-SLO, while also showing that repeatability is influenced by factors such as fixational instability, imaging modality, retinal eccentricity and disease state [7,8,9]. However, repeatability studies for AO-derived vascular metrics have been comparatively limited. Prior AO-based vascular studies have generally focused on younger subjects with normal ocular characteristics, limiting their generalizability to older populations [10]. The repeatability of AO-derived retinal vascular metrics in older adults remains less well characterized, even though aging eyes frequently exhibit reduced fixation stability, increased ocular aberrations, and media opacities that can influence quantitative imaging outcomes [11,12]. Cataracts and other lens opacities may affect AO imaging not only by reducing retinal image contrast, but also by compromising the wavefront-sensing signal used to guide AO correction. Increased scatter and reduced transmission through an opacified lens can reduce the quality of the wavefront-sensor signal, potentially impairing wavefront estimation and limiting the effectiveness of AO correction [11]. Few studies have directly examined the repeatability of AO-derived retinal vascular metrics in older subjects and those with cataracts.

Flood-illuminated AO imaging represents an AO imaging approach in which full-field retinal images are acquired in a single exposure rather than by point-by-point scanning [13]. Flood AO systems have been used to quantify retinal vascular metrics and have demonstrated sensitivity to vascular alterations in glaucoma [4,14] and other ocular disease contexts [15]. While these systems are readily available and can quantify vascular change in age-related ocular disease, the repeatability of flood AO-derived retinal vascular measurements in older adults and in the presence of cataracts has not been systematically evaluated. This is important because older adults represent a large and clinically relevant population for retinal vascular imaging, but age-related media opacities may reduce image quality and affect measurement reliability. In this study, we evaluated the repeatability of retinal vascular metrics obtained in a larger older-adult cohort (40 participants, 66 eyes) with lens opacities by using the commercially available rtx1 flood-illuminated adaptive optics retinal camera (Imagine Eyes, Orsay, France).

## 2. Materials and Methods

### 2.1. Study Design

This prospective observational study was conducted at the Department of Ophthalmology and Visual Sciences at the University of Maryland, Baltimore (UMB). All procedures adhered to the tenets of the Declaration of Helsinki and were approved by the UMB Institutional Review Board (IRB).

### 2.2. Participants

Individuals were screened and recruited from the faculty practice of the Department of Ophthalmology and Visual Sciences. Informed consent was obtained from all participants. No monetary compensation was given in exchange for participating in the study. Inclusion criteria included age 18 or older, ability to understand, and provide informed consent and comply with the imaging protocol. Participants were excluded from the study if they had visually significant media opacities that precluded spectral-domain OCT imaging or if the patient was pregnant or nursing. A total of 47 subjects consented to participate and were enrolled in the study. Seven subjects did not complete the study due to an inability to maintain fixation on the internal target (*n* = 4), drowsiness (*n* = 2), or withdrawal of consent (*n* = 1). Consequently, 40 subjects completed the study and were included in the final analysis.

### 2.3. Clinical Assessment

Subjects underwent a baseline ophthalmological examination, including slit lamp examination, applanation tonometry, gonioscopy, and ophthalmoscopy. Nuclear sclerosis was graded at the slit lamp by a single experienced examiner using a modified LOCS II-style clinical scale [16]. In this subjective scale, trace cataracts were coded as 0.5, whole-number grades such as 1+ were coded as 1.0, and intermediate grades such as 1–2+ were coded as 1.5. Prior to imaging, 34 participants were dilated using 1% tropicamide.

### 2.4. Acquisition of Flood-Adaptive Optics Imaging

All images were acquired using a commercially available flood AO retinal camera (rtx1, Imagine Eyes, Orsay, France). As per protocol, the device was manually adjusted to center the participant’s retinal arteries within the 4° × 4° imaging window. After the camera’s height was adjusted by the operator for the patient’s comfort, the live AO-corrected image visible on the display screen was used to adjust the device until the lumen and outer wall of the patient’s retinal arteries were in focus. Participants maintained fixation on an internal target to ensure accurate measurement of the region of interest. A stack of 40 images was then acquired over a 2 s interval.

**Figure 1:**
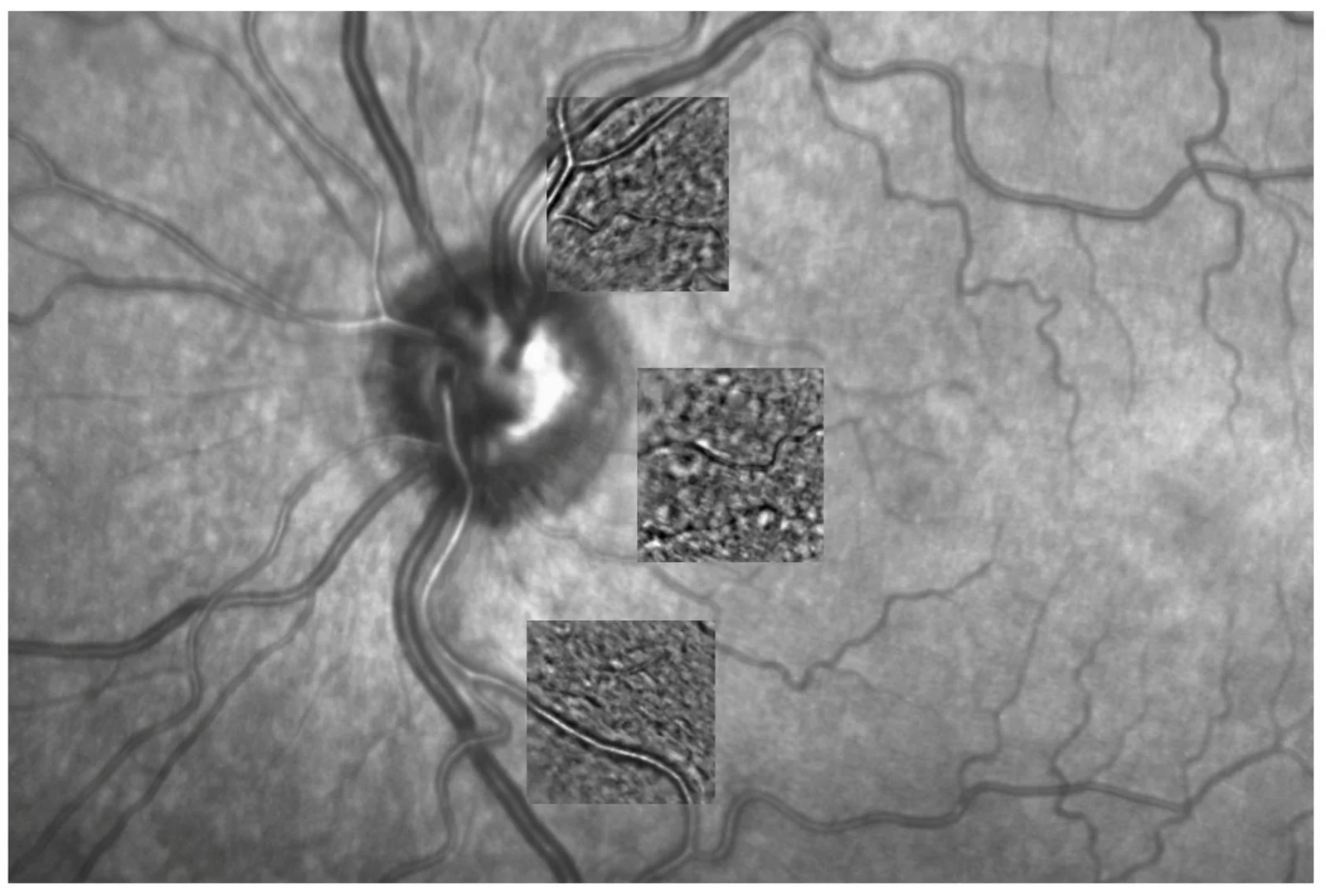
Retinal imaging locations relative to the optic disk. Three adaptive optics imaging regions are shown: superior (**top**), temporal (**middle**), and inferior (**bottom**) to the optic disk.

The protocol consisted of imaging at three distinct locations with visible retinal vessels superior, inferior, and temporal to the optic disk (Figure 1). To assess intrasession repeatability, three consecutive images were acquired at each of these locations. Participants then sat back from the device for a 10 s rest period before being repositioned at the instrument. A second imaging session was then performed, with one image taken at each of the three locations. This entire repositioning procedure was repeated once more to yield a total of three separate imaging sessions within the same time frame.

### 2.5. Image Grading and Analysis

**Figure 2:**
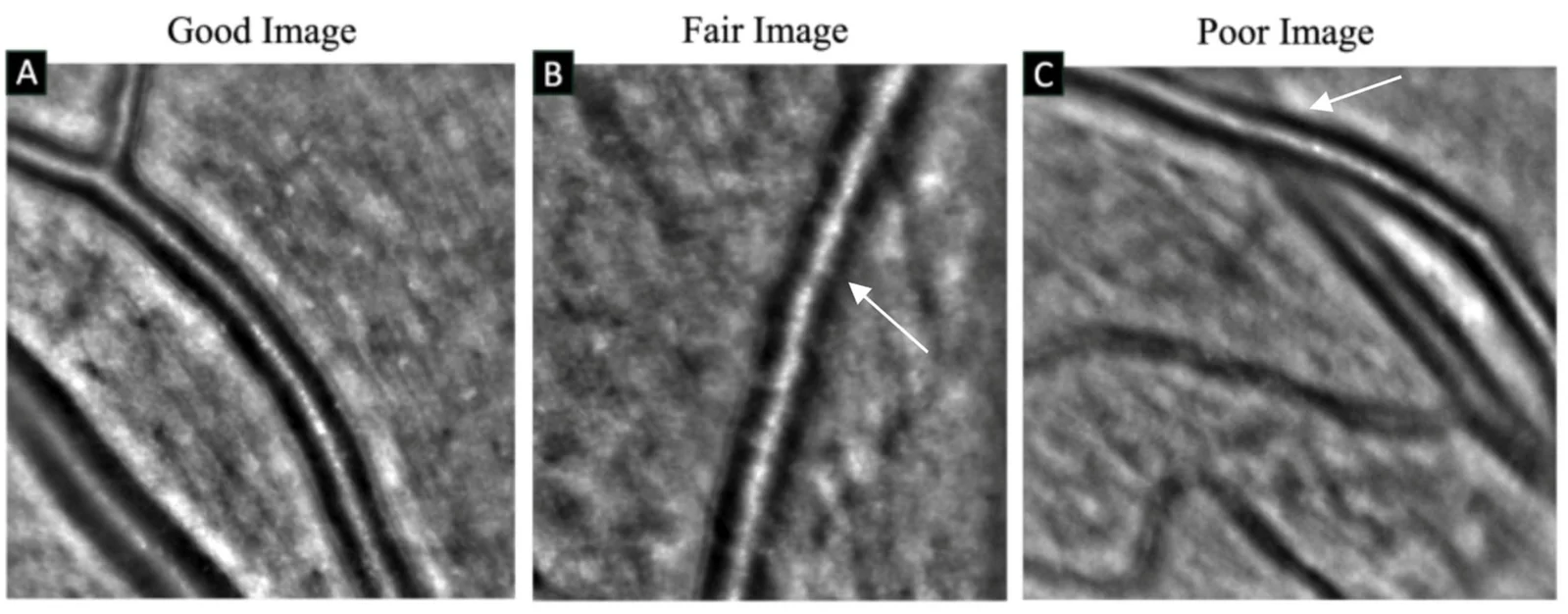
Representative adaptive optics images illustrating the three image quality grades used in this study: (**A**) Good. (**B**) Fair. (**C**) Poor. Arrows depict areas where vessel walls are not clearly visible.

After image acquisition, image sets from each retinal location were aligned using the i2k Retina AO software Version 3.1 (Dual Align; Imagine Eyes, Orsay, France) to ensure that vessel measurements were obtained from the same anatomical segment across repeated captures. Images affected by excessive motion artifact (of the five acquired per location) could not be reliably registered by the software. In these cases, measurement locations were manually reselected, and analyses were limited to inter-grader agreement. Each image was evaluated for image quality as ‘Good’, ‘Fair’, or ‘Poor’. Good images were defined as having both outer vessel walls clearly visible and defined. Fair images were defined as having only one outer vessel wall clearly visible and defined. Poor images were those where neither outer vessel wall could be distinguished, or where the image was blurry (Figure 2).

Quantitative analysis of the retinal vasculature was performed using custom-built software in MATLAB R2025a (MathWorks, Natick, MA, USA). The primary metrics of interest measured from each vessel were the total diameter (TD), lumen diameter (LD), wall cross-sectional area (WCSA), and wall-to-lumen ratio (WLR) (Figure 1). The total diameter of the vessel was defined as the lumen diameter + left wall + right wall. Clear, well-defined areas of the vessel without tortuosity were chosen for analysis. All images were analyzed independently by two experienced, masked graders (YP, KE). A total of 100% of the images were analyzed by both graders.

**Figure 3:**
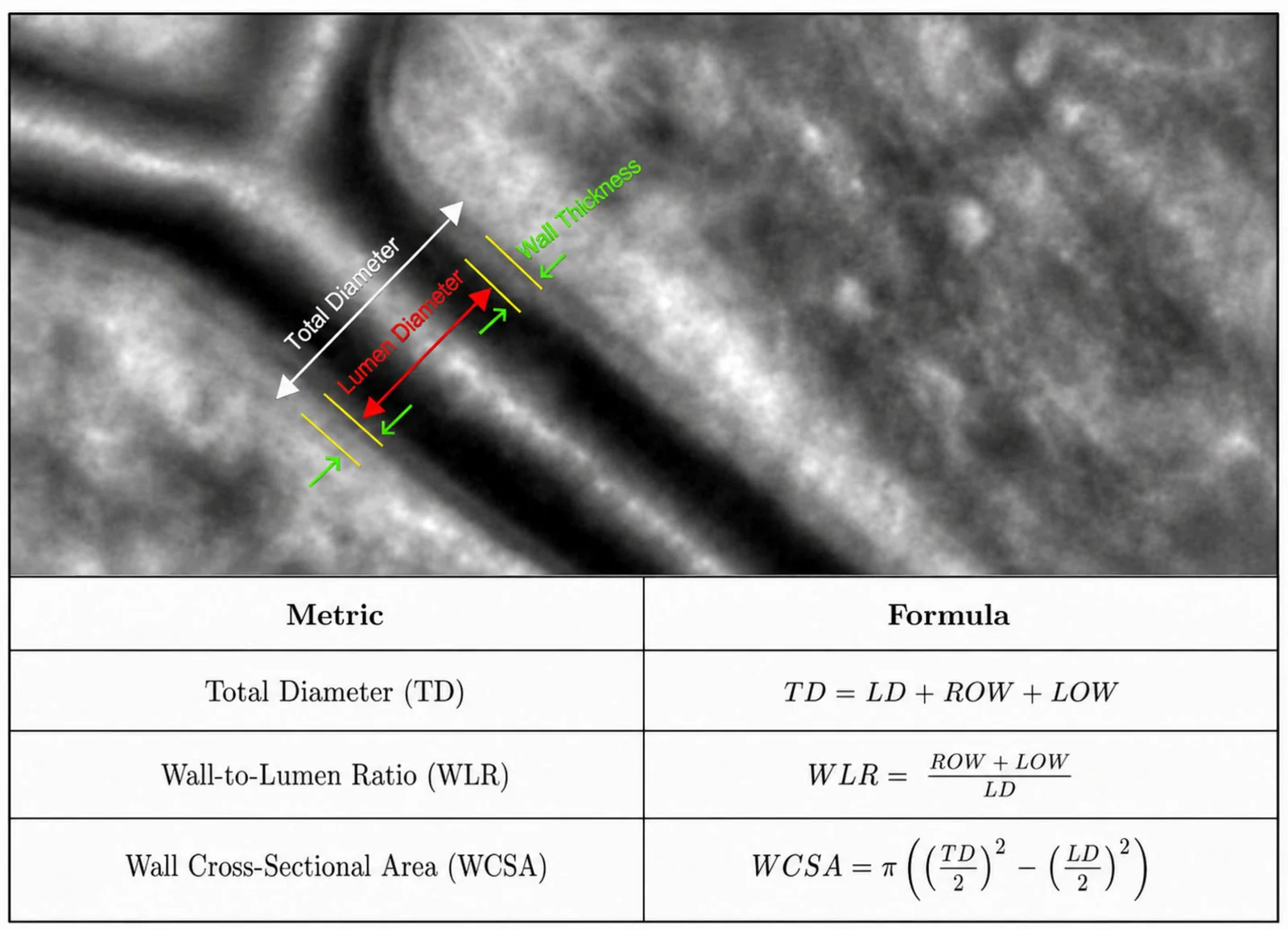
Image of a retinal artery overlaid with measurement annotations: lumen diameter (LD, red), wall thickness (green), which includes the right outer wall (ROW) and left outer wall (LOW), and total diameter (white). The accompanying table defines the key metrics: total diameter (TD), wall-to-lumen ratio (WLR), and wall cross-sectional area (WCSA) and presents their mathematical formulas.

A semi-automated measurement protocol was developed and is outlined in Figure 3, with the steps described below. A line segment was manually drawn perpendicular to the vessel’s axis, after which the software automatically identified the lumen and vessel wall boundaries. The operator refined the segmentation as needed to match visible vessel margins. The software then calculated vessel metrics at the initial line position and at nine additional locations spaced one pixel apart along the vessel axis. Graders drew line segments across the exact same segment of the vessel. Measurements from all ten locations were averaged to yield a single value per metric for each image.

For superior and inferior images, both vessel wall and lumen measurements were obtained. In temporal images, where vessel walls were difficult to visualize due to smaller vessel diameter, analysis was limited to lumen diameter only.

### 2.6. Statistical Analysis

The repeatability of the measurements was assessed by calculating the ICC values. All statistical analyses were conducted using SAS Version 9.4 (SAS Institute, Cary, NC, USA). Our analyses accounted for within-participant correlation because both eyes from some participants were included. The standard deviation (SD) of repeated vessel measurements was estimated using mixed-effects models that included random effects for participant, eye, session, and grader. This allowed the model to account for correlation between fellow eyes, repeated imaging sessions within the same eye, and repeated measurements by graders. Intrasession and intersession ICCs were calculated based on the ratio of variance components derived from repeated measurements.

The total standard deviation (SD) reflects the overall variability in the measurements, including variation due to differences between patients, eyes, sessions, and graders. Our analysis separately accounts for the repeatability between graders, within sessions (intrasession), and between sessions (intersession). For inter-grader agreement between two graders on the same image, the inter-grader standard deviation was calculated as the variability between repeated observations of the same image. The intraclass correlation coefficient (ICC) for inter-grader agreement was calculated as 1 minus the proportion of variance due to graders relative to the total variance: ICC_grader_ = 1 − (inter-grader SD)^2^/(total SD)^2^.

For intrasession repeatability, the intrasession SD reflects variability between measurements of two images of the same vessel taken during the same session by different graders. The intrasession ICC was then computed as 1 minus the ratio of the intrasession variance to the total variance; that is, ICC_intra_ = 1 − (intrasession SD)^2^/(total SD)^2^.

Similarly, the intersession standard deviation was calculated as the variability between images of the same vessel from two different sessions of the same person, assessed by different graders. The intersession ICC was computed as 1 minus the ratio of the intersession variance to the total variance; that is, ICC_inter_ = 1 − (intersession SD)^2^/(total SD)^2^.

The ICC was estimated based on 950 images, each measured twice. We also examined the ICC for intrasession and intersession repeatability across subgroups defined by age, cataract grade, and image quality. Finally, we assessed the relationship between age, the presence of cataract, the presence of an intraocular lens (IOL), cataract grade, and anatomical region and image quality, adjusting for repeated measures within participants using generalized estimating equations.

## 3. Results

### 3.1. Patient Demographics

66 eyes of 40 subjects were included in the final analysis. Table 1 illustrates the demographics of the enrolled subjects and the image quality distribution. All patients were above 50 with 21 patients being 50–65 years old. The majority of eyes had some form of cataract (44 eyes) and were non-glaucomatous (48 eyes). There was a near equal representation of African Americans (47.5%) and Caucasians (45%), and there were more females than males in the cohort (60%). Only 2.1% of images were graded as “poor” with the remainder being good or fair quality.

### 3.2. Agreement in Vessel Metrics of One Image of the Same Vessel by Two Graders (Inter-Grader)

ICC values were categorized as per convention into excellent (>0.9), good (0.75–0.9), moderate (0.5–0.75), and poor (<0.5) [17]. Inter-grader agreement was calculated for all three regions (the superior region, the inferior region, the temporal region) and is shown in Table 2. For images taken at the superior region, the inter-grader agreement for the lumen diameter, total diameter, and WCSA was excellent with an ICC_grader_ of 0.994, 0.990, and 0.952, respectively. Images taken at the inferior region showed similar numbers, with an ICC_grader_ value of 0.984 for lumen diameter, 0.985 for total diameter, and 0.902 for WCSA. Lastly, the ICC_grader_ was 0.974 for the lumen diameter at the temporal region.

### 3.3. Agreement in Vessel Metrics of Two Images in the Same Session by Two Graders (Intrasession)

The intrasession repeatability of each metric of vessels at the superior, inferior and temporal region is represented by the ICC_intra_ in Table 3. Lumen diameter showed excellent repeatability across all locations with the highest being an ICC_intra_ of 0.993 in the superior region. Total diameter and WCSA showed excellent repeatability in the superior and inferior regions but not the temporal region.

### 3.4. Agreement in Vessel Metrics of Two Images in the Same Session by Two Graders (Intersession)

Table 3 depicts the intersession repeatability of the vessel metrics at the three locations. It also includes the standard deviation (SD) between images from two sessions within one patient. LD showed excellent repeatability across all regions. Both WCSA and TD showed excellent repeatability across the superior and inferior regions but not the temporal region, with the superior region showing better repeatability for both metrics compared to the inferior region. WLR showed moderate repeatability in the superior region and good repeatability in the inferior region.

### 3.5. Analysis of Effect of Image Grade on Inter-Grader Agreement

Table 4 displays inter-grader agreement for images across regions, stratified by image quality grade. At the superior region, LD, TD, and WCSA had excellent ICC_grader_ values for both “Good” and “Fair” quality images. WLR showed good repeatability for “Good” images (ICC_grader_ = 0.860) and moderate repeatability for “Fair” images (ICC_grader_ = 0.550). “Poor” images had excellent repeatability for LD but had poor ICC_grader_ values for the other metrics. The inferior region showed similar trends to the superior region for “Good” and “Fair” images, with “Good” images having higher ICC_grader_ values for LD, WCSA and WLR. However, “Poor” images had excellent ICC_grader_ values for LD, TD, and WCSA and good ICC_grader_ values for WLR. For the temporal region, “Good” and “Fair” images had excellent repeatability for LD with ICC_grader_ values of 0.980 and 0.970, respectively. ‘Poor’ images had good repeatability for LD with an ICC_grader_ value of 0.764. It is important to note that the ICC estimates for the poor-quality image subgroup should be interpreted cautiously because the subgroup size was small. We also investigated the effect of dilation on inter-grader agreement and repeatability as shown in Table S1 (see Supplementary Material).

### 3.6. Analysis of Relationship Between Image Grade and Various Predictors (Age, Presence of Cataract, Cataract Grade, Region)

Table 5 shows the analysis of the relationship between image grade and various predictors, where the counts in this table represent image-level observations. Age showed no significant association with image quality. The proportion of images rated as good quality was 38% in participants aged 50–59, 49% in those aged 60–69, and 31% in those aged 70–79 (*p* = 0.072). Cataract and IOL status, as well as cataract grade, were not significantly associated with image quality (*p* = 0.43 and *p* = 0.25, respectively). Image region was the only variable significantly associated with image quality (*p* < 0.0001). Images from the temporal region had the lowest percentage of good ratings (12%), compared to 56% and 55% in the superior and inferior regions, respectively. Most temporal images were rated as fair (86%), with only 12% rated as good. In contrast, the superior and inferior regions had similar distributions, with over half of the images rated as good and approximately 42% rated as fair.

### 3.7. Intrasession and Intersession Repeatability Stratified by Age and Cataract Grade

The intrasession and intersession repeatability analysis of images were stratified by age (Table 6). Participants were stratified into three age groups: 50–59 years (*n* = 11), 60–69 years (*n* = 17), and 70–78 years (*n* = 12).

In the superior region, all age groups demonstrated excellent repeatability for lumen diameter (LD) and total diameter (TD), with the 50–59 age group achieving the highest ICC_inter_ values (0.994 and 0.992, respectively). The 50–59 and 60–69 age groups showed excellent repeatability for wall cross-sectional area (WCSA) at the superior region, while the 70–78 year group demonstrated good repeatability (ICC_intra_ = 0.838). The inferior region showed similar trends to the superior region, with the oldest age group again demonstrating slightly lower ICC values compared to the other age groups. In the temporal region, all age groups achieved excellent repeatability for LD, with the 60–69 year group showing the highest ICC_inter_ (0.984), followed by the 50–59 year group (0.953) and the 70–78 year group (0.950).

Participants were also categorized by cataract grade (Table 7): ≤1 (*n* = 16), 1.5–2.5 (*n* = 20), and 3 (*n* = 6). In the superior region, nearly all cataract grades demonstrated excellent ICC values for LD, TD, and WCSA, except for WCSA in the ≤1 group (ICC_intra_ = 0.893). Wall-to-lumen ratio (WLR) showed moderate repeatability for cataract grades ≤ 1 (ICC_intra_ and ICC_inter_ = 0.617) and 1.5–2.5 (ICC_intra_ and ICC_inter_ = 0.648), but excellent repeatability for grade 3 cataracts (ICC_intra_ and ICC_inter_ = 0.929). The inferior region demonstrated similar trends, with LD and TD showing excellent repeatability across all cataract grades. Although grade 3 cataracts had lower ICC values for LD and TD compared to other grades, repeatability remained excellent. In the temporal region, LD achieved excellent repeatability across all cataract grades, with grade 3 cataracts showing the lowest ICC_inter_ (0.949).

## 4. Discussion

We conducted the first study that examines the effect of age and cataract presence on the repeatability of vascular metrics in an adaptive optics system. We found that vascular metrics are highly reproducible across age groups and cataract grades using a commercially available flood AO system. This is the first such analysis in a large cohort of subjects that is representative of an office-based population. This work will enable further analysis of vascular metrics in older populations with some lens opacity. Given that three of the four major causes of blindness are both age-related and are associated with alterations in the retinal vasculature, this work is needed, as image degradation with AO systems is a major concern. Zhang et al. conducted a similar study assessing AO-SLO performance on photoreceptor imaging in age-matched healthy controls stratified by lens opacity. They found that AO-SLO could acquire useful cone images in most older eyes, but lens opacity still meaningfully influenced image quality [18].

Whereas prior studies on repeatability of vascular metrics in OCT angiography have been conducted and shown high reproducibility, few such studies have been done using adaptive optics systems. Notably, both approaches have potential utility in glaucoma diagnosis and management. Recent work has shown that vascular differences between glaucoma patients and healthy controls can be measured and detected using spectral-domain optical coherence tomography (SD-OCT) [19] and the same commercial flood AO device (rtx1, Imagine Eyes, Orsay, France) [4,14]. However, no prior studies have evaluated vascular metric repeatability in adaptive optics-based devices, where lens opacities can potentially cause degradation in image quality and hence affect repeatability of vascular metrics. Previous repeatability studies using this flood AO-based system have largely been limited to younger cohorts, with minimal inclusion of older individuals or those with cataracts. Soliman et al. demonstrated excellent intra-operator and inter-operator repeatability for lumen diameter and vessel walls [20]. However, that investigation included a relatively small sample (11 subjects with no ocular disease) and did not specify the age range of subjects.

Media opacities like cataract are known to reduce image quality and skew quantitative outcomes [21,22,23,24]. This repeatability study fills this critical gap, demonstrating that flood AO imaging provides excellent repeatability of retinal vascular parameters in older patients with eyes of mild to moderate cataracts. This group has previously shown high repeatability of vascular metrics for AO-SLO and AO-OCT in a smaller cohort of older patients, and this work shows this more robustly in a larger cohort of subjects [10].

In our cohort, measurements obtained before and after dilation showed good to excellent reproducibility. Repeatability was generally similar across dilation status, with the main difference observed for inferior total diameter, where the ICC increased from 0.881 before dilation to 0.984 after dilation with 1% tropicamide. Other measures and regions showed comparable repeatability before and after dilation. While no studies have directly evaluated the effect of mydriasis on AO flood imaging, OCTA and AO-SLO studies have reported improved image quality and reduced motion artifacts after dilation [25,26].

We demonstrated high intersession repeatability for lumen and total diameter, with their ICCs exceeding 0.98. This indicates that both image-level and rater-level variability were minimal for these direct vessel measurements, supporting their robustness as quantitative endpoints. In contrast, derived metrics showed lower repeatability, with intersession ICCs of 0.956 and 0.899 for WCSA in the superior and inferior regions, respectively, and 0.673 and 0.790 for WLR. This discrepancy likely reflects the compounding of measurement errors that occur when multiple dimensions are combined to calculate metrics.

Repeatability was generally higher for the larger superior and inferior vessels than for the smaller temporal vessels. For lumen diameter, intersession ICCs were 0.991 in the superior region and 0.983 in the inferior region, compared with 0.971 in the temporal region. Wall-derived metrics could not be assessed in the temporal region due to inadequate wall visualization. This is consistent with our prior work on OCTA showing that smaller vessels are more susceptible to proportional measurement error [27].

This is particularly relevant to AO imaging, where there are concerns about whether devices such as flood AO systems can reliably resolve and quantify smaller structures. The relatively larger caliber of the peripapillary vessels assessed in this study may therefore represent an inherent advantage for vascular applications of flood AO imaging, where the size of the target structure may buffer against the measurement variability that can affect smaller cellular-level features.

This study demonstrates high repeatability of retinal artery measurements using the commercial flood AO system. However, there are some limitations that should be addressed. Our imaging protocol was focused on the peripapillary vessels, and so further work will require analysis of other vessels, particularly in the macular region. We included analysis of smaller temporal vessels, but these frequently lacked clearly visualized walls and so analysis for that location was only limited to lumen diameter. As a result, repeatability of wall-derived metrics could not be assessed for these smaller temporal vessels. Another limitation to note is that because a subset of eyes in this cohort had glaucoma, absolute vascular measurements may have been influenced by glaucoma-related vascular remodeling [14]. However, because this study focused on repeatability rather than disease-specific vascular differences, glaucoma eyes were retained to reflect a real-world older clinical population. Finally, the poor-quality image subgroup was small because only 2.1% of images were graded as poor. Therefore, ICC estimates for poor-quality images may be unstable and should be interpreted accordingly. This subgroup was retained as an exploratory analysis because poor-quality images represent the most challenging acquisition condition and provide useful information about the limits of measurement reliability under suboptimal imaging conditions.

## 5. Conclusions

This study demonstrates that retinal vascular metrics measured using the rtx1 flood adaptive optics system (Imagine Eyes, Orsay, France) exhibit excellent inter-grader, intrasession, and intersession repeatability in an older population. Lumen diameter and total diameter showed excellent repeatability across age groups, cataract grades, and imaging sessions. Derived metrics such as WCSA and WLR showed acceptable but lower repeatability. This was likely due to compounded measurement variability. Overall, these findings support the short-term repeatability of flood adaptive optics imaging for quantitative assessment of retinal vascular structure in older adults and in eyes with mild to moderate cataracts. Further longitudinal studies are needed to determine whether these measurements remain reproducible over longer follow-up intervals and are suitable for monitoring vascular change over time.

## Figures and Tables

**Table 1:** Demographic and clinical characteristics of enrolled subjects and image quality distribution.

Demographics		No. of Patients
Sex	Female	24 (60%)
	Male	16 (40%)
Race	African American	19 (47.5%)
	Caucasian	18 (45.0%)
	Asian	3 (7.5%)
Age	50–59 years	11 (27.5%)
	60–69 years	17 (42.5%)
	70–78 years	12 (30.0%)
Diagnoses		No. of Eyes
Lens Status	Cataract	44 (66.7%)
	Phakic	14 (21.2%)
	Pseudophakic	8 (12.1%)
Glaucoma	Yes	18 (27.3%)
	No	48 (72.7%)
Dilation	Yes	56 (84.5%)
	No	10 (15.5%)
Image Quality		No. of Images
Good		393 (41.4%)
Fair		537 (56.5%)
Poor		20 (2.1%)

**Table 2:** Inter-grader agreement of retinal vessel metrics across superior, inferior, and temporal imaging regions. ICC values with 95% confidence intervals are reported for each measure.

Location	Measure	Mean	SD (Images)	SD (Raters)	ICC_grader_ (95% CI)
Superior	Lumen Diameter (μm)	76.3	20.70	1.610	0.994 (0.993, 0.995)
	Total Diameter (μm)	102.0	25.75	2.551	0.990 (0.988, 0.992)
	WCSA (μm^2^)	3567.2	1703.52	372.835	0.952 (0.942, 0.962)
	WLR (unitless)	0.3	0.10	0.061	0.595 (0.523, 0.667)
Inferior	Lumen Diameter (μm)	90.4	20.28	2.589	0.984 (0.980, 0.987)
	Total Diameter (μm)	116.3	21.56	2.629	0.985 (0.982, 0.988)
	WCSA (μm^2^)	42,861	1338.94	419.644	0.902 (0.881, 0.922)
	WLR (unitless)	0.3	0.08	0.041	0.765 (0.720, 0.811)
Temporal	Lumen Diameter (μm)	38.3	11.10	1.780	0.974 (0.969, 0.980)

**Table 3:** Intrasession and intersession repeatability of retinal vessel metrics across superior, inferior, and temporal imaging regions. SD and ICC values are reported for measurements within and between sessions.

Location	Measure	Mean	SD (1)	SD (2)	ICC_intra_ (3)	SD (4)	ICC_inter_ (5)
Superior	Lumen Diameter (μm)	75.8	20.82	1.771	0.993	1.974	0.991
	Total Diameter (μm)	101.2	25.94	2.384	0.992	2.500	0.991
	WCSA (μm^2^)	3541.2	1715.26	357.138	0.957	361.344	0.956
	WLR (unitless)	0.3	0.10	0.056	0.673	0.056	0.673
Inferior	Lumen Diameter (μm)	90.5	20.51	2.419	0.986	2.650	0.983
	Total Diameter (μm)	116.6	21.77	2.558	0.986	2.691	0.985
	WCSA (μm^2^)	4281.5	1340.75	406.523	0.908	426.422	0.899
	WLR (unitless)	0.3	0.09	0.039	0.796	0.039	0.790
Temporal	Lumen Diameter (μm)	38.2	11.13	1.750	0.975	1.900	0.971

SD (1): Standard deviation between multiple images (includes variation due to patient, eye, session, and grader). SD (2): Standard deviation between multiple images within sessions. ICC_intra_ (3): Intraclass correlation coefficient between multiple images within sessions. SD (4): Standard deviation between images from two sessions on same patient. ICC_inter_ (5): Intraclass correlation coefficient between images from two sessions on same patient.

**Table 4:** Inter-grader agreement of vessel metrics stratified by image quality grade across superior, inferior, and temporal imaging regions.

Location	Image Quality	Measure	Mean	SD (Images)	SD (Raters)	ICC (95% CI)
Superior	Good	Lumen Diameter (μm) (*n* = 175)	83.3	18.34	1.489	0.993 (0.992, 0.995)
		Total Diameter (μm) (*n* = 175)	108.4	21.94	1.760	0.994 (0.992, 0.995)
		WCSA (μm^2^) (*n* = 175)	3898	1485.58	327.762	0.951 (0.941, 0.962)
		WLR (unitless) (*n* = 175)	0.310	0.07	0.025	0.860 (0.831, 0.889)
	Fair	Lumen Diameter (μm) (*n* = 131)	68.9	19.36	1.757	0.992 (0.990, 0.994)
		Total Diameter (μm) (*n* = 101)	93.7	27.26	3.012	0.988 (0.985, 0.990)
		WCSA (μm^2^) (*n* = 101)	3129	1897.76	413.245	0.953 (0.942, 0.963)
		WLR (unitless) (*n* = 101)	0.316	0.09	0.063	0.550 (0.473, 0.628)
	Poor	Lumen Diameter (μm) (*n* = 6)	36.3	10.570	1.674	0.975 (0.969, 0.980)
		Total Diameter (μm) (*n* = 6)	54.5	9.642	7.923	0.325 (0.226, 0.424)
		WCSA (μm^2^) (*n* = 6)	1295	557.339	557.339	0.000 (−0.111, 0.111)
		WLR (unitless) (*n* = 6)	0.560	0.309	0.298	0.074 (−0.036, 0.184)
Inferior	Good	Lumen Diameter (μm) (*n* = 179)	89.0	20.28	1.200	0.996 (0.996, 0.997)
		Total Diameter (μm) (*n* = 175)	116.2	21.77	1.769	0.993 (0.992, 0.995)
		WCSA (μm^2^) (*n* = 175)	4335	1332.37	347.012	0.932 (0.918, 0.947)
		WLR (unitless) (*n* = 175)	0.304	0.08	0.027	0.878 (0.853, 0.904)
	Fair	Lumen Diameter (μm) (*n* = 135)	92.0	20.26	3.726	0.966 (0.959, 0.974)
		Total Diameter (μm) (*n* = 111)	116.9	21.11	3.604	0.971 (0.964, 0.977)
		WCSA (μm^2^) (*n* = 111)	4243	1309.99	510.759	0.848 (0.817, 0.879)
		WLR (unitless) (*n* = 111)	0.295	0.09	0.056	0.634 (0.567, 0.700)
	Poor	Lumen Diameter (μm) (*n* = 10)	91.4	21.04	1.992	0.991 (0.989, 0.993)
		Total Diameter (μm) (*n* = 7)	109.6	25.28	2.231	0.992 (0.990, 0.994)
		WCSA (μm^2^) (*n* = 7)	3751	1955.66	475.648	0.941 (0.928, 0.954)
		WLR (unitless) (*n* = 7)	0.271	0.08	0.033	0.825 (0.790, 0.860)
Temporal	Good	Lumen Diameter (μm) (*n* = 39)	47.2	14.38	2.05	0.980 (0.975, 0.984)
	Fair	Lumen Diameter (μm) (*n* = 271)	37.1	10.03	1.73	0.970 (0.964, 0.977)
	Poor	Lumen Diameter (μm) (*n* = 4)	35.1	3.74	1.82	0.764 (0.717, 0.810)

**Table 5:** Association between demographic and clinical variables and image quality grade across all imaging sessions.

Variable	Level	Number (%) Poor	Number (%) Fair	Number (%) Good	*p*-Value
Age Group	50–59 (*n* = 279)	3 (1%)	179 (61%)	106 (38%)	0.072
	60–69 (*n* = 433)	9 (2%)	211 (49%)	213 (49%)	
	70–79 (*n* = 238)	8 (3%)	156 (66%)	74 (31%)	
Cataract/IOL	Neither (*n* = 177)	0 (0%)	81 (46%)	96 (54%)	0.43
	IOL, no cataract (*n* = 86)	1 (1%)	35 (41%)	50 (58%)	
	Cataract, no IOL (*n* = 587)	17 (3%)	345 (59%)	225 (38%)	
	Both (*n* = 14)	0 (0%)	14 (100%)	0 (0%)	
Cataract Grade	0.5 or 1 (*n* = 259)	6 (2%)	140 (54%)	113 (44%)	0.25
	1.5, 2, or 2.5 (*n* = 283)	11 (4%)	184 (65%)	88 (31%)	
	3 (*n* = 90)	2 (2%)	49 (54%)	39 (43%)	
Region	Superior (*n* = 324)	6 (2%)	131 (42%)	175 (56%)	<0.0001
	Inferior (*n* = 312)	10 (3%)	135 (42%)	179 (55%)	
	Temporal (*n* = 314)	4 (1%)	271 (86%)	39 (12%)	

**Table 6:** Intrasession and intersession repeatability of retinal vessel metrics stratified by age group across superior, inferior, and temporal imaging regions.

Location	Age Group	Measure (Units)	Mean	SD (1)	SD (2)	ICC_intra_ (3)	SD (4)	ICC_inter_ (5)
Superior	50–59 (*n* = 11)	Lumen Diameter (μm)	70.9	23.33	1.681	0.995	1.733	0.994
		Total Diameter (μm)	93.0	30.28	2.715	0.992	2.715	0.992
		WCSA (μm^2^)	2959	1907.12	290.388	0.977	290.388	0.977
		WLR (unitless)	0.282	0.07	0.049	0.523	0.049	0.523
	60–69 (*n* = 17)	Lumen Diameter (μm)	84.0	18.69	1.475	0.994	1.899	0.990
		Total Diameter (μm)	112.1	22.59	1.865	0.993	2.056	0.992
		WCSA (μm^2^)	4257	1653.00	381.719	0.947	392.422	0.944
		WLR (unitless)	0.318	0.07	0.030	0.839	0.036	0.768
	70–78 (*n* = 12)	Lumen Diameter (μm)	66.6	16.874	2.303	0.981	2.356	0.981
		Total Diameter (μm)	89.9	19.225	2.773	0.979	2.995	0.976
		WCSA (μm^2^)	2874	933.679	375.800	0.838	376.748	0.837
		WLR (unitless)	0.361	0.157	0.091	0.663	0.091	0.663
Inferior	50–59 (*n* = 11)	Lumen Diameter (μm)	89.2	27.30	2.348	0.993	2.465	0.992
		Total Diameter (μm)	112.5	27.52	1.931	0.995	2.111	0.994
		WCSA (μm^2^)	3678	1481.18	329.635	0.950	358.035	0.942
		WLR (unitless)	0.261	0.09	0.048	0.676	0.048	0.676
	60–69 (*n* = 17)	Lumen Diameter (μm)	95.5	17.82	2.593	0.979	2.806	0.975
		Total Diameter (μm)	122.9	18.78	2.188	0.986	2.553	0.982
		WCSA (μm^2^)	4767	1175.08	345.177	0.914	366.688	0.903
		WLR (unitless)	0.301	0.07	0.029	0.821	0.029	0.821
	70–78 (*n* = 12)	Lumen Diameter (μm)	84.2	16.35	2.184	0.982	2.583	0.975
		Total Diameter (μm)	110.1	18.20	3.516	0.963	3.516	0.963
		WCSA (μm^2^)	4081	1232.32	563.098	0.791	578.817	0.779
		WLR (unitless)	0.335	0.11	0.041	0.854	0.043	0.833
Temporal	50–59 (*n* = 11)	Lumen Diameter (μm)	38.4	10.21	1.98	0.962	2.22	0.953
	60–69 (*n* = 17)	Lumen Diameter (μm)	41.0	12.04	1.40	0.986	1.52	0.984
	70–78 (*n* = 12)	Lumen Diameter (μm)	33.9	9.24	1.99	0.954	2.07	0.950

SD (1): Standard deviation between multiple images (includes variation due to patient, eye, session, and grader). SD (2): Standard deviation between multiple images within sessions. ICC_intra_ (3): Intraclass correlation coefficient between multiple images within sessions. SD (4): Standard deviation between images from two sessions on same patient. ICC_inter_ (5): Intraclass correlation coefficient between images from two sessions on same patient.

**Table 7:** Intrasession and intersession repeatability of retinal vessel metrics stratified by cataract grade across superior, inferior, and temporal imaging regions.

Location	Cataract Grade	Measure (Units)	Mean	SD (1)	SD (2)	ICC_intra_ (3)	SD (4)	ICC_inter_ (5)
Superior	≤1 (*n* = 16)	Lumen Diameter (μm)	83.5	19.60	1.283	0.996	1.393	0.995
		Total Diameter (μm)	112.2	21.60	3.150	0.979	3.150	0.979
		WCSA (μm^2^)	4119	1512.45	494.427	0.893	494.427	0.893
		WLR (unitless)	0.303	0.08	0.051	0.617	0.051	0.617
	1.5–2.5 (*n* = 20)	Lumen Diameter (μm)	64.1	17.78	1.943	0.988	2.173	0.985
		Total Diameter (μm)	87.1	22.79	2.433	0.989	2.433	0.989
		WCSA (μm^2^)	2691	1328.05	295.039	0.951	324.607	0.940
		WLR (unitless)	0.347	0.15	0.087	0.648	0.087	0.648
	3 (*n* = 6)	Lumen Diameter (μm)	69.6	14.80	1.857	0.984	2.889	0.962
		Total Diameter (μm)	90.0	20.61	2.281	0.988	3.788	0.966
		WCSA (μm^2^)	2637	1337.54	220.345	0.973	346.404	0.933
		WLR (unitless)	0.293	0.10	0.025	0.929	0.025	0.929
Inferior	≤1 (*n* = 16)	Lumen Diameter (μm)	97.6	15.78	1.921	0.985	1.921	0.985
		Total Diameter (μm)	123.7	14.44	1.760	0.985	1.870	0.983
		WCSA (μm^2^)	4813	1181.53	347.888	0.913	378.564	0.897
		WLR (unitless)	0.300	0.09	0.028	0.901	0.028	0.894
	1.5–2.5 (*n* = 20)	Lumen Diameter (μm)	85.5	22.31	2.500	0.987	2.904	0.983
		Total Diameter (μm)	111.8	26.33	3.393	0.983	3.445	0.983
		WCSA (μm^2^)	4239	1603.54	492.236	0.906	523.461	0.893
		WLR (unitless)	0.328	0.10	0.055	0.670	0.056	0.657
	3 (*n* = 6)	Lumen Diameter (μm)	78.0	21.71	4.163	0.963	4.163	0.963
		Total Diameter (μm)	98.1	28.47	3.666	0.983	3.685	0.983
		WCSA (μm^2^)	3222	1719.16	389.584	0.949	389.584	0.949
		WLR (unitless)	0.300	0.09	0.041	0.786	0.041	0.786
Temporal	≤1 (*n* = 16)	Lumen Diameter (μm)	39.0	8.43	1.25	0.978	1.44	0.971
	1.5–2.5 (*n* = 20)	Lumen Diameter (μm)	37.9	12.16	2.30	0.964	2.43	0.960
	3 (*n* = 6)	Lumen Diameter (μm)	35.6	6.31	1.11	0.969	1.42	0.949

SD (1): Standard deviation between multiple images (includes variation due to patient, eye, session, and grader). SD (2): Standard deviation between multiple images within sessions. ICC_intra_ (3): Intraclass correlation coefficient between multiple images within sessions. SD (4): Standard deviation between images from two sessions on same patient. ICC_inter_ (5): Intraclass correlation coefficient between images from two sessions on same patient.

## Data Availability

Dataset available on request from the authors.

## Supplementary Materials

The following supporting information can be downloaded at: https://www.mdpi.com/article/10.3390/bioengineering13080852/s1. Table S1: Inter-grader agreement of vessel metrics for dilated patients across superior, inferior, and temporal imaging regions.

## Abbreviations

The following abbreviations are used in this manuscript:

AO	Adaptive Optics
AO-SLO	Adaptive Optics Scanning Laser Ophthalmoscopy
AO-OCT	Adaptive Optics Optical Coherence Tomography
SD-OCT	Spectral-Domain Optical Coherence Tomography
LD	Lumen Diameter
TD	Total Diameter
WLR	Wall-to-lumen ratio
WCSA	Wall cross-sectional area
ROW	Right Outer Wall
LOW	Left Outer Wall
ICC	Intraclass Correlation Coefficient
SD	Standard Deviation
IOL	Intraocular lens
UMB	University of Maryland, Baltimore
IRB	Institutional Review Board
