# Supplementary material for: Quantitative Retinal Vascular Imaging Using Flood-Illuminated Adaptive Optics in Eyes with Lens Opacities
Source: Bioengineering (Basel). 2026 Jul 23;13(8):852. doi: 10.3390/bioengineering13080852 (PMC13509802; doi:10.3390/bioengineering13080852)
Supplement: Supplementary file 1 [file bioengineering-13-00852-s001.zip › bioengineering-4385402-supplementary.pdf]

Article

Quantitative Retinal Vascular Imaging Using Flood-Illuminated Adaptive Optics in Eyes with Lens Opacities

Yash Porwal, Kenaan Elbash, Marlon Diaz, Emily Butler, Saige Oechsli, Laurence Magder, Andrew J. Bower and Osamah Saeedi *

Department of Ophthalmology and Visual Sciences, University of Maryland, Baltimore, MD 21201, USA; yporwal@som.umaryland.edu (Y.P.); kelbash@som.umaryland.edu (K.E.); marlon.diaz@som.umaryland.edu (M.D.); emily.butler@som.umaryland.edu (E.B.); soechsli@som.umaryland.edu (S.O.); lmagder@som.umaryland.edu (L.M.); abower@som.umaryland.edu (A.J.B.)

* Correspondence: osaeedi@som.umaryland.edu; Tel.: +667-214-1232

Supplementary Data

We also investigated the effect of dilation on inter-grader agreement and repeatability, as shown in Table A1. In non-dilated patients, ICCs for lumen diameter were excellent across all regions, while total diameter showed excellent repeatability in the superior region and good repeatability in the inferior region (ICC = 0.881). In contrast, dilated patients demonstrated higher repeatability for total diameter in the inferior region (ICC = 0.984) and maintained excellent ICCs for lumen diameter across all regions. Total diameter measurements in dilated eyes were also excellent in both the superior and inferior regions.

Table S1. Inter-grader agreement of vessel metrics for dilated patients across superior, inferior, and temporal imaging regions.

Location	Condition	Measure	Mean	SD (Images)	SD (Raters)	ICC (95% CI)
Superior	Dilated	Lumen Diameter (μm)	73.5	19.70	1.705	0.993 (0.991, 0.994)
		Total Diameter (μm)	98.7	24.69	2.750	0.988 (0.985, 0.990)
		WCSA (μm²)	3391.2	1646.09	402.621	0.940 (0.927, 0.953)
		WLR (unitless)	0.327	0.10	0.068	0.565 (0.489, 0.640)
Inferior	Dilated	Lumen Diameter (μm)	89.5	19.82	2.733	0.981 (0.977, 0.985)
		Total Diameter (μm)	114.7	21.98	2.742	0.984 (0.981, 0.988)
		WCSA (μm²)	4294.2	1411.15	403.909	0.918 (0.901, 0.936)
		WLR (unitless)	0.309	0.08	0.041	0.756 (0.708, 0.803)
Temporal	Dilated	Lumen Diameter (μm)	38.0	10.83	1.64	0.977 (0.972, 0.982)
